# Aromatic Amino Acid Mutagenesis at the Substrate Binding Pocket of *Yarrowia lipolytica* Lipase Lip2 Affects Its Activity and Thermostability

**DOI:** 10.1155/2014/382581

**Published:** 2014-08-13

**Authors:** Guilong Wang, Zimin Liu, Li Xu, Yunjun Yan

**Affiliations:** Key Laboratory of Molecular Biophysics of the Ministry of Education, College of Life Science and Technology, Huazhong University of Science and Technology, No. 1037, Luoyu Road, Wuhan 430074, China

## Abstract

The lipase2 from *Yarrowia lipolytica* (YLLip2) is a yeast lipase exhibiting high homologous to filamentous fungal lipase family. Though its crystal structure has been resolved, its structure-function relationship has rarely been reported. By contrast, there are two amino acid residues (V94 and I100) with significant difference in the substrate binding pocket of YLLip2; they were subjected to site-directed mutagenesis (SDM) to introduce aromatic amino acid mutations. Two mutants (V94W and I100F) were created. The enzymatic properties of the mutant lipases were detected and compared with the wild-type. The activities of mutant enzymes dropped to some extent towards *p*-nitrophenyl palmitate (*p*NPC16) and their optimum temperature was 35°C, which was 5°C lower than that of the wild-type. However, the thermostability of I100F increased 22.44% after incubation for 1 h at 40°C and its optimum substrate shifted from *p*-nitrophenyl laurate (*p*NPC12) to *p*-nitrophenyl caprate (*p*NPC10). The above results demonstrated that the two substituted amino acid residuals have close relationship with such enzymatic properties as thermostability and substrate selectivity.

## 1. Introduction

As a group of industrial enzymes of great importance, lipases (EC 3.1.1.3) catalyze not only the hydrolysis of triglycerides at water-oil interface, but also esterification and transesterification in nonaqueous phase, such as organic-solvent medium [1]. Thus, lipases have been applied in many fields, for example, enrichment of polyunsaturated fatty acids, synthesis of valuable esters, enzymatic preparation of biodiesel, and resolution of chiral compounds [2].

Among the reported lipases, lipase2 from* Yarrowia lipolytica* has been extensively studied. The researches mainly focus on expression and purification, immobilization and applications, and protein engineering for improving thermostability [3]. According to our previous research, this enzyme was overexpressed in* Pichia pastoris* with a protein yield of 7.62 g L^−1^ in a 10 L-scale fermentor after 145 h culture [4]. The immobilization on magnetic nanoparticles in reverse micelles system of enzymes has achieved good catalytic performance for enrichment of polyunsaturated fatty acids [5]. It exhibits a promising prospect in application.

Moreover, the structure of YLLip2 has been reported in detail by Bordes et al. [6]. Its structure is highly homologous to the known structures of the fungal lipase family:* Rhizomucor miehei* lipase (RML),* Rhizopus niveus* lipase (RNL), and* Thermomyces lanuginose* lipase (TLL) [6]. According to the classification of Pleiss et al., 3D-structure alignment of YLLip2 and the above-mentioned three lipases showed that they all shared hydrophobic crevice-like substrate binding sites [7]. For YLLip2, the crevices consist of T88,V94, I98, I100, F129, L163, P190,V232,V235, P236, and Y241 corresponding to S82, W88, L92, F94, F111, L145, P177,V205, L208, P209, and F215 in RML; T83, A89, I93, F95, F112, L146, P178, V206, V209, P210, and F215 in RNL; and S82, W88, L92, F112, L146, P173, V202, L205, P206, and F213 in TLL. Theoretically, the amino acids at the corresponding positions are structurally equivalent. However, from the knowledge of amino acid chemistry, V94 in YLLip2 is quite different from W88 in RML and TLL. Similarly, I100 in YLLip2 is distinct from F94 in RML and TLL and F95 in RNL. In fact, V and I are from aliphatic series whereas W and F are aromatic amino acids. In the classification of amino acids, the aromatic amino acids are classified into a special group due to their large side chains and the unique benzene ring structure. Thus, they can form many interactions with the surrounding microenvironment, such as hydrophobic interactions, *π*-*π* stacking, and *π*-cation interactions. Some studies have been performed to illustrate the functions of aromatic residue in the binding pocket of TLL [8, 9]. The side chain size and hydrophobicity of V94 varied greatly from that of W88. Moreover, I100 and F94 (F95) have similar hydrophobicity but different side chain sizes.

Therefore, in order to explore their role in determining enzymatic properties of YLLip2, we chose V94 and I100 for mutation, and they were, respectively, substituted by W and F using SDM. Two mutant lipases were created. Subsequently, their enzymatic properties were examined and compared with the wild-type, and the reasons of the variance were further discussed.

## 2. Materials and Methods

### 2.1. Bacterial Strains, Plasmids, and Reagents

*Escherichia coli* DH5*α* (Invitrogen, USA) competent cells were used for transformation and vector amplification. pMD18-T-flip2 (T-vectors harboring full-length YLLip2 gene), plasmid pJME803 (a kind gift from Jean-marc Nicaud, CNRS, Institut Micalis, Domaine de Vilvert, France), and pINA1297 (a kind gift from Catherine Madazak, INRA, UMR1319 Micalis, Domaine de Vilvert, Jouy-en-Josas, France) were stored in our laboratory. Strain* Y. lipolytica* JMY1212 (a kind gift from Jean-marc Nicaud, CNRS, Institut Micalis, Domaine de Vilvert, France) for protein expression was also stored in our lab. Plasmid extraction kit and DNA purification kit were purchased from Omega (USA). PrimeSTAR HS DNA polymerase, restriction endonucleases, and DNA Ligation Kit were purchased from Takara (Dalian, China). QuikChange Site-Directed Mutagenesis Kit was purchased from Agilent Technologies Inc. (CA, USA).* p*-Nitrophenyl esters were bought from Sigma-Aldrich (St. Louis, USA). All other reagents used were of analytical grade and commercially available from Sinopharm Chemical Reagent Co., Ltd. (Shanghai, China).

### 2.2. Plasmid Construction, Site-Directed Mutagenesis, and Yeast Transformation

First, in order to conveniently introduce N-terminal 6 × histidine fusion tag, plasmid pYL was constructed as follows: pJME803 and pINA1297 were doubly digested with ClaI and BamHI; for the former plasmid, long fragments harboring selection markers and integration sequence were recovered; for the latter plasmid, short fragments harboring the hybrid promoter hp4d with XPR2pre signal peptide were recovered. Then, the two fragments were ligated together and transformed into* E. coli* DH5*α* competent cells and plated on LB solid plates containing 40 ug/mL kanamycin. The construction was confirmed by sequencing and defined as pYL. Consequently, lipase expression vector was constructed: the DNA sequence encoding mature YLLip2 was amplified by polymerase chain reaction (PCR) using the primer YLLip2-f (5′-cctgccgttctggcc*caccaccaccaccaccac*gtgtacacctctacc-3′) and YLLip2-r (5′-cctcctaggttagataccaca-3′). Two restriction sites (BglI and AvrII, underlined) and 18 bp sequence (in italics) encoding N-terminal 6 × histidine tag were introduced. The fragments were double-digested and inserted into pYL digested with the same enzymes. The recombinant plasmid was defined as pYL-H6-mYLLip2. In this plasmid, lipase expression was driven by the hp4d promoter and its secretion was mediated by the XPR2pre signal region [10]. Site-directed mutagenesis was performed using the recombinant plasmid as template by QuikChange Site-Directed Mutagenesis Kit. For V94W, the primers were V94W-f (5′-gaacccactctctggaggactggataaccgacatccgaatcat-3′) and V94W-r (5′-atgattcggatgtcggttatccagtcctccagagagtgggttc-3′). For I100F, the primers were I100F-f (5′-ccgacatccgattcatgcaggctcc-3′) and I100F-r (5′-ggagcctgcatgaatcggatgtcgg-3′). All mutations were confirmed by DNA sequencing. For* Y*.* lipolytica* transformation, the plasmids were digested with NotI; long fragments harboring target genes were recovered and then transformed into the* Y. lipolytica *JMY1212 competent cells according to the method described by Xuan et al. [11]. After transformation, the mixture was plated on YNBDcasa solid medium (6.7% YNB, 1% D-dextrose, 0.2% casein acid hydrolysates, and 1.5% agar) [12]. After 48-hour cultivation at 28°C, the colonies were transferred to YNBT solid agar (6.7% YNB, 1% tributyrin, and 1.5% agar) for activity assay. The colonies with clear halos were identified as positive clones for further study [13]. The integration of lipase gene to the yeast chromosome was confirmed by PCR using the genomic DNA of the selected positive clones.

### 2.3. Protein Expression and Purification

For protein expression, the positive clones of wild-type YLLip2 and mutants were inoculated in 5 mL liquid YPD (1% yeast extract, 2% peptone, and 2% D-dextrose) medium for overnight cultivation and then transferred to liquid YT_2_D_5_ medium (1% yeast extract, 2% peptone, and 5% D-dextrose) for enzyme production [6]. After cultivation for 72 h at 28°C, the fermentation broth was centrifuged at 12,000* g* at 4°C and supernatant was collected for protein purification using one-step immobilized metal affinity chromatography. The eluted protein was dialyzed against 50 mmol phosphate buffer solution (PBS, pH 8.0) to remove imidazole and glycerol. The purity of lipase was checked by SDS-polyacrylamide gel electrophoresis (SDS-PAGE). Protein concentration was determined by Bradford method using bovine serum albumin (BSA) as standard [14].

### 2.4. Lipases Substrate Assay

Lipase activity was determined by measuring the release of pNP using the chromometer method with some modifications [15]. Lipase substrate range and specific activity were determined under the standard conditions using* p*-nitrophenyl esters with various acyl chain lengths:* p*-nitrophenyl acetate (C2),* p*-nitrophenyl butyrate (C4),* p*-nitrophenyl caprylate (C8),* p*-nitrophenyl caprate (C10),* p*-nitrophenyl laurate (C12),* p*-nitrophenyl-myristate (C14), and* p*-nitrophenyl palmitate (C16). For activity assay, first, the reaction mixture consisted of 20 *μ*L 10 mmol* p*-nitrophenyl esters dissolved in ethanol, 970 *μ*L 50 mmol PBS (pH 8.0), which were incubated at 40°C; then 10 ul of properly diluted purified enzyme solutions was added and hydrolysis started. Ten minutes later, the reaction was stopped on ice bath. Then, the optic density was measured via UV-Vis spectra at 405 nm. Lipase activity was calculated according to the molar extinction coefficients of* p*-nitrophenol of 17311.6 M^−1^ cm^−1^ (pH 8.0). One unit of enzyme activity was defined as the amount of enzyme needed to liberate 1 *μ*mol of* p*-nitrophenol per minute.

### 2.5. Effect of Temperature and pH on Lipase Activity and Thermostability

To determine the optimum temperature, enzyme activities were measured at temperatures ranging from 25°C to 55°C in 50 mmol PBS (pH 8.0) using* p*-nitrophenyl palmitate (C16) as substrate. For thermostability assay, the enzyme solutions were incubated at 40°C with different duration and residual activities were detected using* p*-nitrophenyl palmitate (C16) as substrate. To test the optimum pH, lipase activity was detected at 40°C in buffers with various pH values (50 mmol acetic acid sodium acetate buffer solution for pH 5.5, 50 mmol PBS for pH 6.0–8.0, and 50 mmol glycerine-NaOH buffer for pH 8.5–9.0).

### 2.6. Lipase Kinetics Assay

Purified lipases were used for kinetic analysis at 40°C using* p*-nitrophenyl palmitate (C16) as substrate. To determine kinetic parameters, the initial reaction rates under various substrate (0.2–1 mmol) concentrations were calculated. The Michaelis-Menton constant (*K*_*m*_) and the maximum velocity (*V*_max⁡_) were determined by Lineweaver-Burk plot.

### 2.7. Three-Dimensional Structure Modeling and Substrate Docking

The 3D structures of the wild-type and mutant enzymes in their open conformation were built by homology modeling using the MODELLER package program (V9.13, http://salilab.org/modeller/) [16]. The crystal structures of RML (pdb: 4TGL), TLL (pdb: 1GT6) in their open conformation and* Aspergillus niger *feruloyl esterase A (AnFaeA.pdb: 1USW) were selected as templates. After energy minimization, molecular docking of* p*NP-esters with lipases was performed by Autodock 4.2 (http://autodock.scripps.edu) in the default parameters and best pose of the enzyme-substrate complex was chosen for analysis. All the structures were visualized by PyMol (http://www.pymol.org). Protein intramolecular interactions were analyzed by the Protein Interactions Calculator (PIC) server (http://pic.mbu.iisc.ernet.in/index.html) [17].

## 3. Results and Discussion

### 3.1. SDS-PAGE of Purified Enzymes and Lipase Activity towards Different pNP-Esters

**Figure 1:**
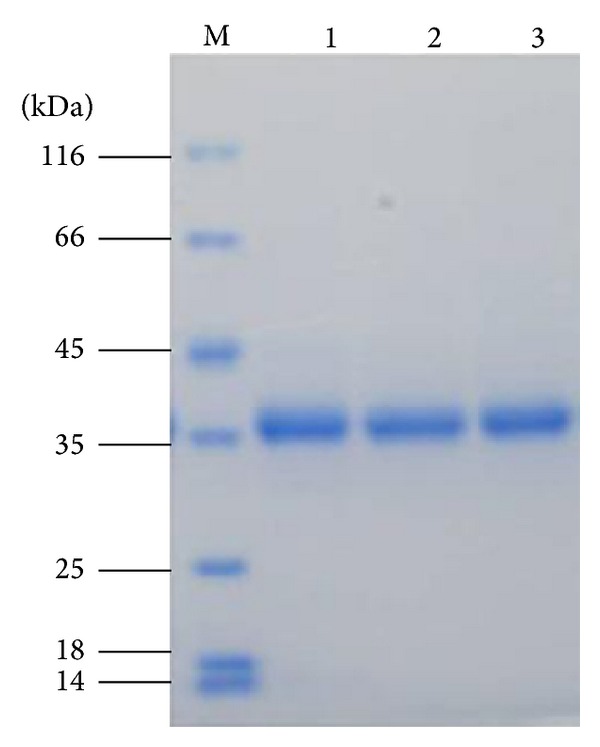
SDS-PAGE of WT and mutant enzymes. M: protein molecular weight marker (Fermentas SM0431). Lane 1–Lane 3 are wild-type V94W and I100F, respectively.

The SDS-PAGE (see Figure 1) results showed that each lipase was purified homogeneity as a single band. The lipases showed a molecular weight of approximate 38 kDa, which is consistent with our previous research [4]. This indicated that the purified enzymes could be subjected to activity assay and kinetic parameters determination. As shown in Table 1, the experimental data demonstrated that wild-type YLLip2 is an enzyme with preference for medium and long chain* p*NP-esters. The substitution of V94 with W did not change chain length selectivity, but the overall activity decreased. V94 occupies a position in YLLip2 where Trp is found in the lipases of RML and TLL. In RML, this Trp may play a role in the interfacial activation [18]; replacement of Trp did not abolish the interfacial activity but changed the substrate penetration [19]. In lipase RNL, this position is A; when it was replaced by W, its activity towards tributyrin, tricaprylin, and triolein decreased [20]. Our results are similar to these results. Another research showed that the substitution of V72 with F in PEL resulted in loss of activity towards* p*NPC16. Both W and F are bulkier amino acids. The increased steric hindrance is a major cause of activity loss [21]. The reduced activity of V94W mutant might also be caused by this effect. However, for V94W, the mutant was not profoundly changed in the relative activity towards various substrates tested compared with the wild-type enzyme. Additionally, V94 is part of the lid shielding the active sites to keep YLLip2 in its closed conformation. The increased side chain size may weaken the lid movement which has negative effect on active sites exposure, thus decreasing the activity [22].

For the other mutant I100F in Table 1, we can see that the optimum substrate of I100F is* p*-nitrophenyl caprate (*p*NPC10) while the optimum substrate of WT is* p*-nitrophenyl laurate (*p*NPC12). Its activity towards middle chain fatty acid* p*NP-esters increased by 4.59% and 48.00% for* p*NPC8 and* p*NPC10 compared with WT enzyme, respectively.

### 3.2. Temperature and pH Profiles of Wild-Type Enzyme and Mutant Enzymes

**Figure 2:**
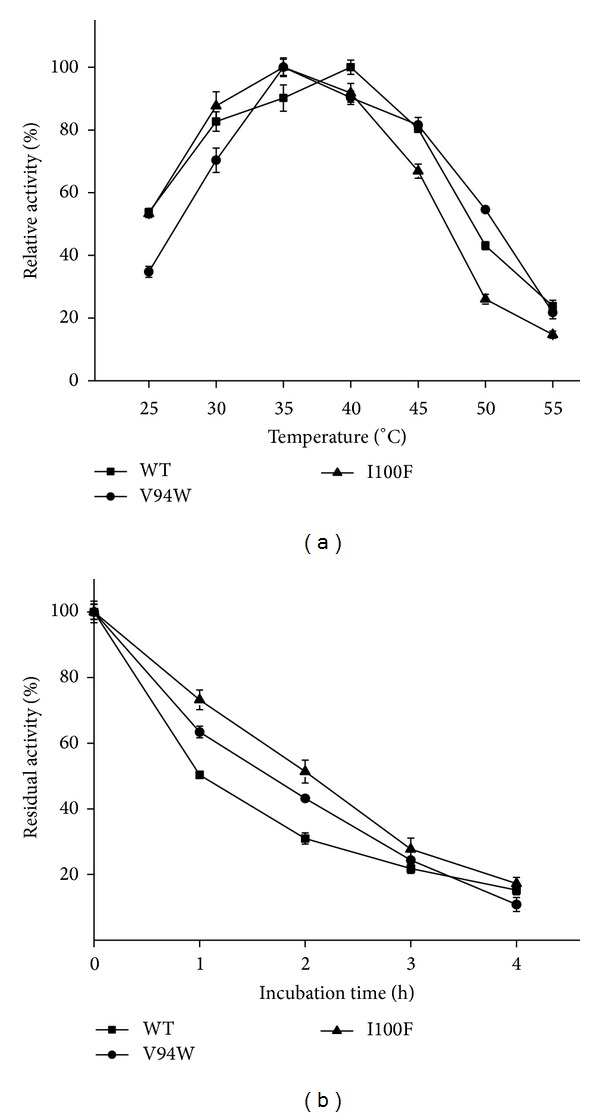
Effect of temperature on lipase activity and thermostability. (a) Effect of temperature on lipase activity. Lipase activity was detected in 50 mmol PBS, pH 8.0 under different temperatures (25–55°C; temperature interval is 5°C). (b) Thermal stability of three lipases. Lipases were incubated at 40°C for different durations and residual activities were measured under standard assay conditions (50 mmol PBS, pH 8.0, and 40°C). The activity at 0 h (nonincubated) was taken as 100%. The values represent the means of three independent experiments (mean ± SD). WT, V94W, and I100F were shown as triangle (▲), circle (●), and square (■).

**Figure 3:**
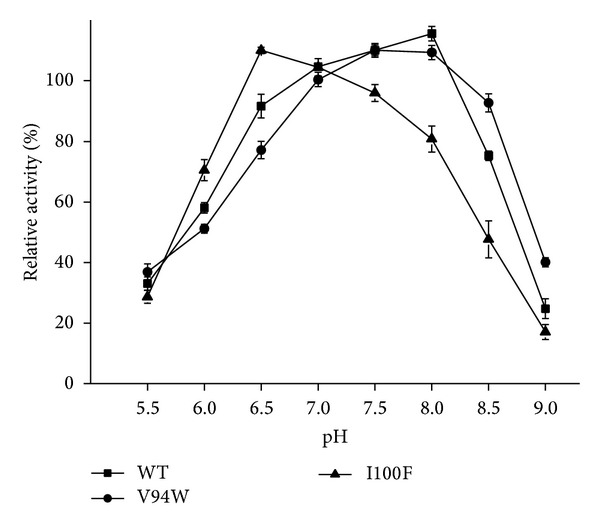
Effect of pH on lipase activity. Lipase activity was detected at 40°C in 50 mmol buffers of various pH values (5.5–9.0; pH value is 0.5). The values represent the means of three independent experiments (mean ± SD). WT, V94W, and I100F were shown as triangle (▲), circle (●), and square (■).

The wild-type enzyme exhibited its maximum activity at 40°C; however, the mutants showed their maximum activity at 35°C (Figure 2). We can speculate that most enzymes are mesophilic enzymes and exhibit good catalytic performance at temperature range from 30 to 45°C. Once the temperature exceeds 50°C, their activities are decreased sharply. The wild-type enzyme retained only 50% residual activity after incubation for 1 h at 40°C; by contrast, the mutants retained 63% and 73% residual activity, respectively. After incubation for 4 hours at 40°C, the residual activity was 15%, 10%, and 17% for WT, V94W, and I100F. The mutants showed better thermostability, among which the best performance came from I100F. V94 is a hydrophobic amino acid. Generally, hydrophobic residuals are packaged inside the protein, but in the opened conformation of YLLip2, these hydrophobic residuals are exposed on the surface of the protein, which makes it tend to repel the solvent. The hydrophobic interactions cause the destabilization of the protein surface. Replacement of V with W reduced the strong interactions and enhanced the stabilizing force of the protein surface, improving thermostability [23]. I100 is at the rim of the acyl binding groove; the hydrophobic interaction is the major force maintaining the local structure of the acyl moiety for efficient catalysis [23]. When I100 was replaced by F, hydrophobicity and hydrophobic interaction sustained. Protein interaction analysis indicated that aromatic-aromatic interactions occurred between F237 and F100, F241 and F100. Therefore, the aromatic stacking force strengthened thermostability to some extent. Similar aromatic cluster was also found in TLL [24]. As for the effect of pH on lipase activity, Figure 3 showed that all the enzymes exhibited good catalytic performance near neutral pH (pH 6.5–8.0). For WT and I100F, the optimum pH is 8.0; however, the relative activity decreased sharply once the pH values exceed 8.0. V94W showed maximum activity at pH 7.0. Moreover, it remained higher relative activity in basic conditions. Perhaps the mutation could stabilize the open conformation of the lipase in basic conditions.

### 3.3. Lipases Kinetics Determination

**Figure 4:**
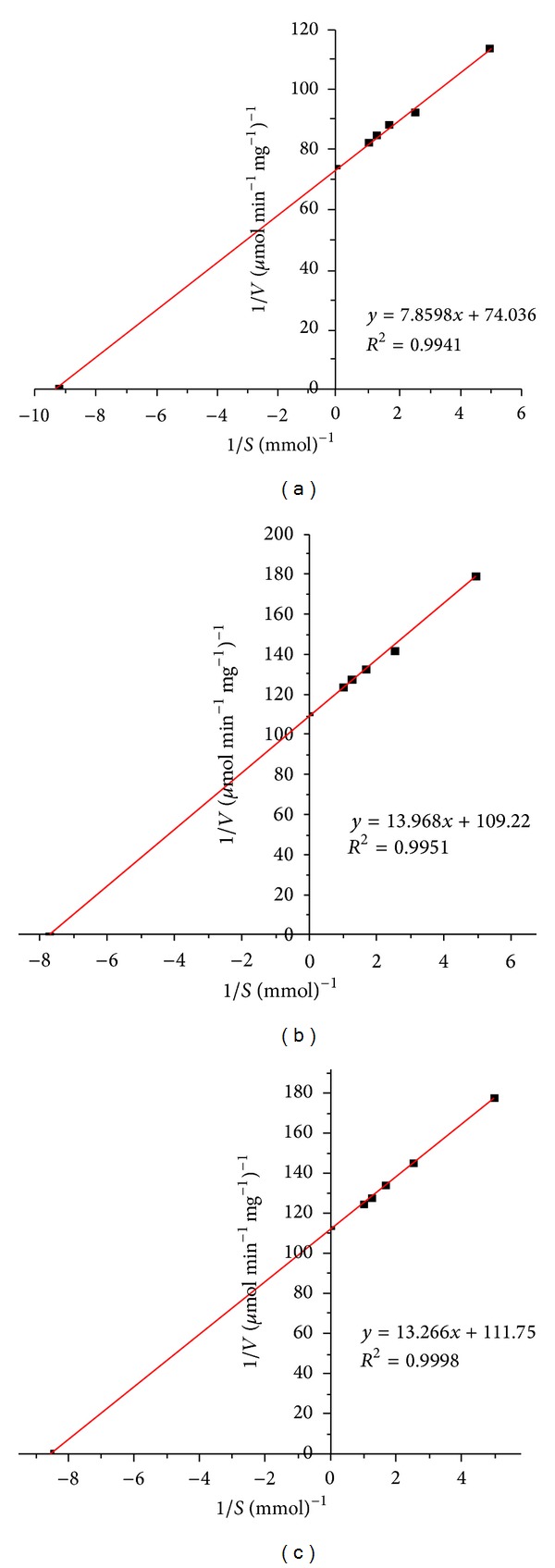
The Lineweaver-Burk plot of the purified lipases. Lipase activity was detected at 40°C in 50 mmol pBS (pH 8.0) using* p*NPC16 with different concentrations (0.2–1.0 mmol) as substrate. (a), (b), and (c) showed the Lineweaver-Burk plot of WT, V94W, and I100F.

The Lineweaver-Burk plot of the purified lipases was presented in Figure 4, and the measured specific activity and kinetic parameters of* p*NPC16 were listed in Table 2. From the table, we can see that the activities of two mutants towards* p*NPC16 were decreased to 75% and 73% of its original one, and their catalytic efficiency decreased to 44% and 40%, respectively. The increased *K*_*m*_ values indicated that the affinity of mutant enzymes to* p*NPC16 also decreased. One reason is that substitution of V with W at position 94 increased the side chain size and enhanced steric hindrance to substrate access to the active site, lowering the activity [21], especially for* p*NP-esters with long fatty acid chain. The other reason is that, for lipases, the enzymes make contact with substrates mainly by hydrophobic interaction. Though W is a hydrophobic residue, it is less hydrophobic than V; thus the decreased hydrophobic interaction caused loss of activity. For I100, the decreased activity resulted from the steric clashes occurring when the long chain fatty acid bended or folded in the acyl groove. The degree of steric clashes for I100F is higher than that of WT because of the increased side chain of F. Thus, the activity of I100 towards* p*NPC16 decreased [25].

### 3.4. Structure Modeling and Molecular Docking of YLLip2 and the Mutants

**Figure 5:**
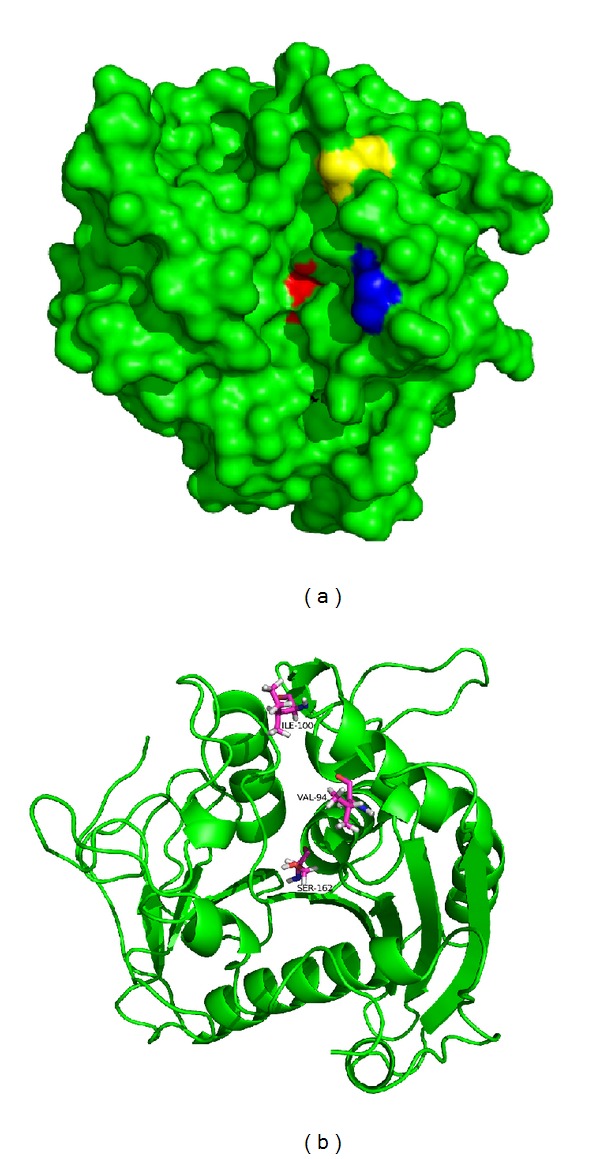
3D structure of the opened conformation of YLLip2. (a) Structure was shown as surface. Ser162 was colored red while Val94 and I100F were colored blue and yellow. (b) Structure was shown as cartoon where Ser162, Val94, and I100F were shown as sticks.

**Figure 6:**
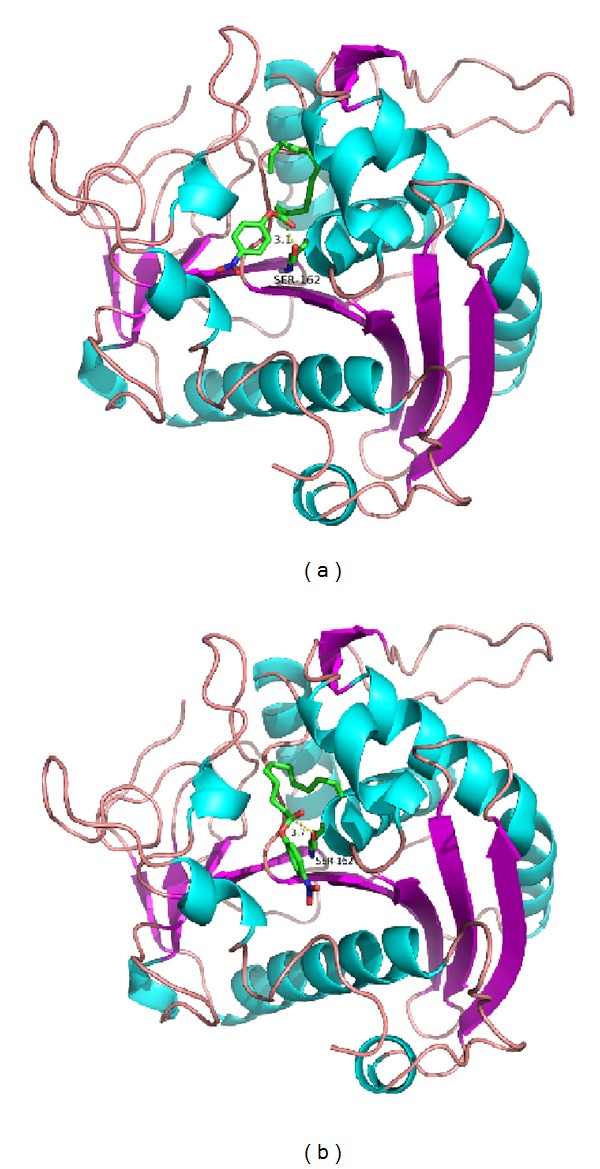
Molecular docking of WT and I100F with* p*NPC12. The distance between O_*γ*_ of S162 and the carbon atom of carbonyl group in* p*NPC12 was measured. (a) WT-*p*NPC12 complex; (b) I100F-*p*NPC12 complex.

The constructed structure of YLLip2 in its open conformation (Figure 5) confirmed to typical *α*/*β* hydrolase fold. It consists of six *α*-helix and eight *β*-sheets. The catalytic triad is formed by Ser162, Asp 230, and His289. Ser162 is exposed during catalysis [6]. Though the crevice-like substrate binding pocket of YLLip2 is on the surface of the enzyme, what should be pointed out is that V94 is located at the extreme edge of the crevice which is very close to the solvent; thus the substrates need to cross the shallow tunnel to access the catalytic center. I100 is at the rim of a groove which accommodates the acyl-chain of* p*NP-esters [26]. The overall structure of mutants was very similar to that of wild-type enzyme except those small local changes at mutation sites. In order to illustrate substrate specificity change,* p*NPC10 was docked to WT and I100, and two optimal poses were generated. We measured the distance of nucleophile attack; the distance between O_*γ*_ of S162 and the carbon atom of carbonyl group in pNPC12 is 3.1 Å of WT-*p*NPC12 complex; however, in I100F-pNPC12 complex, the distance is 3.7 Å (Figure 6). The attack distance affects the molecular collision during reaction, so the activity of I100F towards* p*NPC12 decreased [23].

## 4. Conclusion

Lipases are a kind of enzymes which have been widely studied, especially for their structure-function relationship, such as the chain length activity and selectivity, stereoselectivity. We found that V94 played a very important role in substrate access. Changes in the side chain size affected the activity towards pNP-esters of different chain length. This is a useful method for those lipase catalyzed bulker substrates which cannot access the active site efficiently. I100F of lip2 with enhanced thermostability was obtained due to the increased aromatic stacking force. This research lays the framework for further engineering lip2 in order to make it more suitable for industrial catalysis.

## Figures and Tables

**Table 1:** Substrate specificity of WT, V94W, and I100F under standard conditions.

Substrates	WT activity (U mg^−1^)	V94W activity (U mg^−1^)	I100F activity (U mg^−1^)
*p*-Nitrophenyl acetate	89.92 ± 1.83	55.43 ± 5.20	43.27 ± 6.61
*p*-Nitrophenyl butyrin	184.10 ± 16.24	114.66 ± 3.05	79.22 ± 3.37
*p*-Nitrophenyl caprylate	336.16 ± 8.12	202.43 ± 8.04	351.62 ± 8.10
*p*-Nitrophenyl caprate	412.95 ± 17.23	305.71 ± 5.75	611.20 ± 8.11
*p*-Nitrophenyl laurate	609.74 ± 26.97	419.81 ± 5.57	474.32 ± 23.84
*p*-Nitrophenyl myristate	398.27 ± 2.82	301.16 ± 4.33	269.09 ± 16.49
*p*-Nitrophenyl palmitate	228.42 ± 5.17	171.20 ± 4.06	166.98 ± 5.48

∗The activities were detected in 50 mmol PBS (pH 8.0) at 40°C. WT means the wild-type enzyme (the amino acid residual in position 94 is V and in 100 is I). The values represent the means of three independent experiments (mean ± SD).

**Table 2:** Specific activity and kinetics parameters of wild-type enzyme and mutant enzymes.

	WT	V94W	I100F
Specific activity (U mg^−1^)	228.42 ± 5.17	171.20 ± 4.06	166.98 ± 5.48
*K*_*m*_ (mmol)	0.106 ± 0.003	0.128 ± 0.002	0.118 ± 0.007
*V*_max⁡_ (*μ*mol min^−1^)	0.0135 ± 0.0009	0.0092 ± 0.0006	0.0089 ± 0.0006
*k*_cat_ (s^−1^)	157 ± 1	107 ± 1	104 ± 1
*k*_cat_/*K*_*m*_ (mmol^−1^ s^−1^)	1491 ± 31	835 ± 16	889 ± 43

∗The activities were tested in 50 mmol pH 8.0 PBS at 40°C. The values represent the means of three independent experiments (mean ± SD).
